# Une tumeur historique de l’épaule

**DOI:** 10.11604/pamj.2017.28.65.13541

**Published:** 2017-09-22

**Authors:** Ilhame Naciri, Baderddine Hassam

**Affiliations:** 1 Service de Dermatologie et Vénérologie, Centre Hospitalier Universitaire IBN SINA, Faculté de Médecine et de Pharmacie, Université Mohammed V, Rabat, Maroc

**Keywords:** Tumeur, historique, épaule, chondrosarcome, Tumor, historical, shoulder, chondrosarcoma

## Abstract

Chondrosarcoma is a malignant tumor of bone of mesenchymal origin which usually occurs in people after age 40 at the level of the pelvis and the shoulder. We report the case of a 65-year old female patient, with no particular past medical history, with impaired general condition, hospitalized for exploration of a large tumor of the right shoulder, evolving over 3 years. Clinical examination showed giant, humpbacked, hard, adherent tumor measuring 44 × 32 cm along its longer axis, with inflammatory signs associated with signs of vasculo-nervous compression. Radiological evaluation showed expansive, epiphyseal, metaphyseal, diaphyseal, heterogeneou, multilobulated, poorly differentiated, calcified tumoral process with complete destruction of the glenohumeral joint that extensively invaded the soft tissues with loco-regional extension. Histological examination of a deep biopsy specimen was in favor of poorly differentiated chondrosarcoma. Staging evaluation objectified bilateral axillary lymphadenopathies and multiple metastatic subpleural and hepatic nodules. The patient died after 2 months. This study highlights the role of awareness campaigns for early diagnosis and management.

## Image en médecine

Le chondrosarcome est une tumeur osseuse maligne d'origine mésenchymateuse, qui survient habituellement après l'âge de 40 ans, et qui siège préférentiellement au niveau du pelvis et de l'épaule. Nous rapportons le cas d'une patiente âgée de 65 ans, sans antécédents pathologiques notables, hospitalisée pour exploration d'une tumeur volumineuse de l'épaule droite, évoluant depuis 3 ans, avec notion d'altération de l'état général. L'examen clinique retrouvait une tumeur géante en dos de chameau, faisant 44 × 32 cm de grand axe, dure, adhérente, avec des signes inflammatoires en regard et des signes de compression vasculo-nerveuse. La mobilité de l'épaule était limitée et douloureuse. Le bilan radiologique objectivait un processus tumoral expansif épiphyso-métaphyso-diaphysaire hétérogène, multilobulé, mal limité, et calcifié, avec une destruction complète de l'articulation gléno-humérale et un important envahissement des parties molles et extension locorégionale. L'examen histologique d'une biopsie profonde était en faveur d'un chondrosarcome peu différentié. Le bilan d'extension révélait des adénopathies axillaire bilatérales et de multiples nodules sous pleuraux et hépatique d'allure métastatiques. L'évolution était fatale au bout de 2 mois. Notre observation souligne l'importance de la sensibilisation de la population pour un diagnostic et une prise en charge précoce.

**Figure 1:**
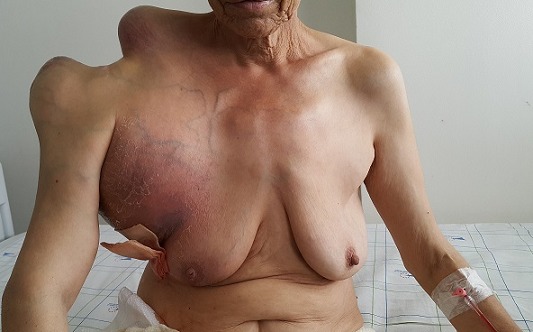
Chondrosarcome géant de l’épaule droite, mesurant 44 × 32 cm

